# Peroneal tendon dislocation in talus fracture and diagnostic value of fleck sign

**DOI:** 10.1007/s00264-020-04534-9

**Published:** 2020-03-17

**Authors:** Ahmed Khalil Attia, Karim Mahmoud, Tarek Taha, Osama AlDahamsheh, Ahmed Hany ElHessy, Ahmad S. AlObaidi, Mohammed M. Mekhaimar

**Affiliations:** 1 Orthopedic Surgery Dept, Hamad General Hospital, P.O. Box 3050, Doha, Qatar; 2 Weil Cornell Medical School-Qatar, Ar-Rayyan, Qatar

**Keywords:** Talus, peroneal tendons, hindfoot, fracture, fleck sign

## Abstract

**Introduction:**

Talus fractures are not uncommon and one of the serious fractures in the foot and ankle. Peroneal tendon dislocation is one of the commonly missed soft tissue injuries which may have significant impact on the outcomes including persistent pain and swelling. They have been reported to be associated with calcaneum as well as talus fractures.

**Aim:**

To report the incidence of peroneal tendon dislocation in talus fracture and the significance of fleck sign in the diagnosis of peroneal tendon dislocation.

**Methods:**

We retrospectively reviewed 93 consecutive talus fractures in the period between 1/1/2011 to 1/11/2018. Inclusion criteria were: The patient underwent open reduction and internal fixation, had pre-operative CT scan that is available for review and three view ankle plain radiographs. Two independent authors review the radiographs for peroneal tendon dislocation, fleck sign and fracture classification, if any. Any dispute was resolved by the senior author.Patient records were reviewed for laterality, age, sex,mode of injury, associated injuries and operative interventions. 50 ankles met the inclusion criteria. 49 were males, mean age was 32.5 year and the predominant mode of injury was a fall from height.

**Results:**

Peroneal tendon dislocation was found in ten patients out of 50 (20%). Risk of dislocation increased with severity of the fracture and neck fractures. Most of the dislocations were missed by surgeons and radiologist, and no additional procedures were done to address such an injury. The Fleck sign had a statistically significant correlation with peroneal tendons dislocations (p=.005)

**Conclusion:**

Peroneal tendons dislocation is associated with as high as 20% of talus fractures. The authors recommend carefully reviewing CT scans by surgeons and radiologists alike to avoid missing such injury and allow for appropriate surgical approach utilization. The Fleck sign is a highly specific radiographic sign that has a statistically significant correlation with PT dislocation and hence we recommend intra-operative assessment of peroneal tendons in patients with the fleck sign.

## Introduction

Talus fractures are rare yet serious fractures that usually necessitate operative intervention with a relatively high risk of complications [1].

Peroneal tendon dislocation is a commonly missed soft tissue injury which may have a significant impact on the outcomes. Available literature suggests that operative management offers better functional outcomes for such injuries [2]. Complications, including persistent pain and swelling, have been reported. It has been associated with other hindfoot injuries such as calcaneal fractures as well as talus fractures [3, 4].

The Fleck sign is a cortical avulsion fracture of the distal tip of the lateral malleolus, also known as rim fracture or fibular sleeve avlusion fracture, van Dijk et al. [5] which is best visualized in internal rotation views. It has been reported to be associated with peroneal tendon dislocation in talus fractures [4].

### Aim

This study aims to determine the prevalence of peroneal tendon dislocation in talar fractures as well as the diagnostic value of the fleck sign.

## Materials and methods

After obtaining approval from the Institutional Review Board (IRB), the authors retrospectively reviewed 93 consecutive talus fractures in the period between January 1, 2011, to January 1, 2018, at a tertiary care center with a well-established foot and ankle service.

Inclusion criteria were:

The patient underwent open reduction and internal fixation, had a pre-operative CT scan that is available for review, had three views of ankle plain radiographs, and the operative report is available on EMR.

**Fig. 1:**
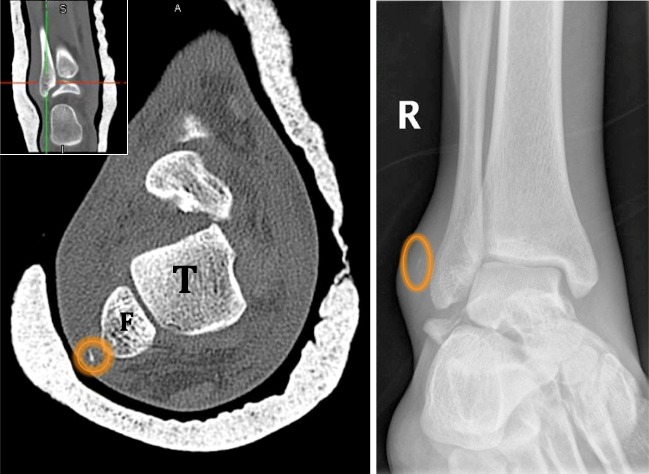
Axial CT cut (Fig. 2a) and internal rotation ankle plain X-ray (Fig. 2b) showing talar neck fracture and fleck sign (circled)

**Fig. 2:**
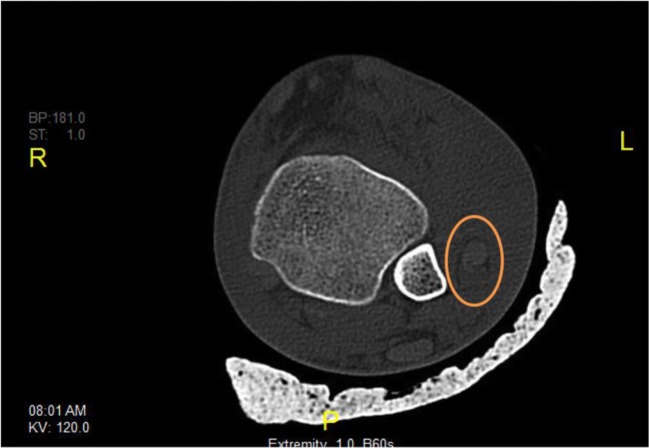
Axial cut from CT scan of a left ankle showing anterior dislocation of peroneal tendons (circled)

Three independent reviewers (AA, KM, and TT) studied the CT scans for peroneal tendon dislocations, plain radiographs and CT scans for the fleck sign (Fig. 1), and fracture classification, if any. Peroneal tendon dislocation was assessed from the axial and coronal CT images according to the criteria of Ho et al. [6]. In the axial image, the peroneal tendon should lie in a triangle formed by the posterolateral margin of the distal fibula, the superior peroneal retinaculum, and the calcaneofibular ligament. In the coronal image, the peroneal tendons should lie within the fibular groove. When dislocated, the tendon lies lateral and anterior to the posterolateral margin of the distal fibula. Figure 2 Subluxations and dislocations were both considered dislocations for the feasibility of the study and statistical analysis. Hawkins classification was used for talus neck fractures. Any dispute was resolved by the senior author (MM). The three reviewers had an almost perfect inter-observer agreement. Patient records were also reviewed for laterality, age, sex, mode of injury, associated injuries and operative interventions, and intra-op findings

A total of 50 talus fractures matched the inclusion criteria. Almost all the patients were males. Mean age was 32.5 years (SD = 9.5 years). The predominant mode of injury was falling from a height followed by road traffic accidents. Most of the fractures were closed while open fractures accounted for 16% of the fractures (Table 1).

**Table 1:** Patient Demographics. Abbreviations: FFH= Fall from height, HO= Heavy object, RTA= road traffic accident, M= Male, F=Female

Mean age (years) (SD)	32.5 (9.5)	
Gender		
M	49	98%
F	1	2%
Mechanism of injury	Number	Percentage
FFH	21	42.0
HO	4	8.0
RTA	11	22.0
Unknown	14	28.0
Open fracture?	Number	Percentage
Closed	42	84.0
Open	8	16.0

Talus fractures were isolated in 23 ankles (46%). Others were associated with other foot and ankle injuries. These injuries were further classified anatomically into ankle malleolar fractures, calcaneal fractures, and midfoot fractures (Table 2).

**Table 2:** Associated foot and ankle injuries

Associated injuries	
Ankle (lat mal/med mal/bimalleolar)	15
Calcaneus	7
Midfoot	6
None	22

Talus fractures were classified according to fracture location into neck, body, and posterior processes. Neck fractures were further classified according to Hawkins classification. Interestingly, a majority of the fractures (72%) were neck fractures, mostly Hawkins type 1.

## Results

Peroneal tendon dislocation was found in ten ankles out of 50 (20%). Almost all dislocations occurred in talar neck fractures, and up to 50% of Hawkins type III and IV talar neck fractures were associated with peroneal tendon dislocation. Risk of dislocation increased with the severity of the fracture (Table 3).

**Table 3:** PT dislocation with talus fracture location

Fracture location	Number	PT dislocation
Neck	36	8/36 (22.2%)
H1	20	1/20 (5%)
H2	7	3/7 (42.8%)
H3	5	2/5 (40%)
H4	4	2/4 (50%)
Body	7	2/7 (28.5%)
Post process	6	0
Total	50	

In terms of concomitant injuries with peroneal tendon dislocation cases, there were three cases of ankle fractures, while one patient had calcaneum fracture. However, the association between those fractures in peroneal tendon dislocation did not reach statistical significance.

The Fleck sign was present in five out of ten PT dislocations both on plain radiographs and CT scans. There was a statistically significant correlation between the fleck sign and PT dislocation (*p* = 0.005).

Table 4 summarizes the clinical details of those who had peroneal tendon dislocation associated with talus fracture.

**Table 4:** Summary of peroneal tendon dislocation cases

Case	Age (years)	Sex	R/L	Mech of injury		Talus fracture	Classification	Associated injuries	Fleck sign	Surgery for talus	Surgery for PT	Diagnosed by radiologist
Case 1	26	M	R	RTA	Closed	Neck	Hawkins II	OCD	Y	ORIF	None	N
Case 2	25	M	R	FFH	Open	Neck	Hawkins IV	Bimalleolar	N	Ex fix + ORIF	None	N
Case 3	34	M	R	HO	Open	Neck	Hawkins IV	Lat mal, heel pad avulsion, navicular, med cuneiform	N	ORIF	anchor repair	Y
Case 4	25	M	L	FFH	Closed	Neck	Hawkins III	none	N	ORIF	anchor repair, Revision (lat mal pain)	N
Case 5	29	M	L	FFH	Closed	Body		calcaneum	Y	ORIF	none	N
Case 6	37	M	L	FFH	Open	Neck	Hawkins III	Medial Mal	Y	ORIF	None	N
Case 7	28	M	L	FFH	Closed	Neck	Hawkins I	Bimalleolar	Y	ORIF	None	N
Case 8	38	M	R	Unknown	Closed	Neck	Hawkins II	Cuboid	Y	ORIF	None	N
Case 9	24	M	R	Unknown	Closed	Neck	Hawkins II	None	N	ORIF	None	N
Case 10	41	M	L	RTA	Closed	Body		None	N	ORIF	Anchor repair	Y

## Discussion

Anatomy of the peroneal tendons is crucial to understanding. Peroneal tendons are contained within a common synovial sheath that splits at the level of the peroneal tubercle. The sheath runs in the retromalleolar sulcus on the fibula that is deepened by a fibrocartilaginous rim. They are covered by superior peroneal retinaculum (SPR) which originates from the posterolateral ridge of the fibula and inserts onto the lateral calcaneus at the peroneal tubercle. The inferior aspect of the SPR blends with the inferior peroneal retinaculum. SPR is thought to be the primary restraint of the peroneal tendons within the retromalleolar sulcus [7]. It is suggested that the axial loading force associated with falling from height leads to disruption of lateral ankle structures including the SPR which consequently leads to peroneal tendon dislocation or instability. Moreover, the presence of the fleck sign has been reported by multiple studies on calcaneal fractures to be predictive of rim fractures [8, 9]. Reviewing the axial and coronal CT images using Ho et al. criteria is suggested. It is also worth mentioning that CT scans may overestimate the prevalence of peroneal tendon dislocation; hence, intraoperative assessment of peroneal tendon stability is paramount for proper management [10, 11].

We have demonstrated that peroneal tendon dislocation is associated with talus fractures and that its incidence is higher in patients with a fleck sign. This finding should compel surgeons to have a high index of suspicion when treating such injuries. The incidence is higher in neck fractures and increases with the severity of the fracture on Hawkins classification [12].

Unfortunately, most of the dislocations (7 out of 10) were missed and no additional procedures were done to address such an injury. Functional outcomes of those patients are not documented on the EMR. The other three patients required additional anchor fixation and, out of those three patients, one patient required revision of peroneal tendon fixation at a later stage. Only two dislocations were diagnosed by the musculoskeletal radiologist, an unfortunate yet common occurrence. Literature reports 75 to 90% of injuries being missed by the radiologists [11, 13, 14].

Approximately one-quarter of the patients who underwent internal fixation for a fracture of the talus had evidence of peroneal tendon dislocation. This figure is comparable with that of dislocation associated with calcaneal fractures [15]. However, associated injuries have not affected the incidence of peroneal tendon dislocation in our study and the association was statistically insignificant.

Peroneal tendon dislocation occurred almost exclusively in talar neck fractures which had a statistically significant correlation. It also occurred more frequently in patients with more severe fractures (Hawkins types III and IV) than in patients with less severe fractures (Hawkins types I and II), although the difference did not reach statistical significance. Our findings are consistent with previous studies of talus fractures, as well as calcaneus fractures [4, 9, 11, 15, 16].

The importance of recognition of peroneal tendon dislocation is that those injuries left untreated are associated with significant morbidity including lateral ankle instability, pain, and clicking. Chronic dislocation is associated with tear of the peroneus brevis due to attrition against the posterolateral ridge of the fibula. This adds to the morbidity of talus fractures and may lead to inferior outcomes of surgical treatment (17–19). Another implication of the presence of peroneal tendon dislocation is that it may affect approach selection for open reduction and internal fixation to allow for examination and treatment of the peroneal dislocation while maintaining maximum exposure. The surgeon may choose to use a posterolateral approach just lateral to the Achilles tendon utilizing the interval between the peronii and flexor halucis muscles in order to tackle the talus fracture as well as peroneal tendon dislocation. The recommended examination technique by Chen et al. is to introduce a Freer elevator within the peroneal tendon sheath followed by application of anterior and lateral forces. Any advancement over the anterior border of the fibula is diagnostic [10]. An anterolateral approach may also be utilized by making an incision in line with the fourth ray between the tibia and fibula, just lateral to the extensor digitorum longus; however, this approach is not ideal for tackling the peroneal tendons. The choice of the lateral approach needs to be carefully considered if a combined anteromedial approach is also indicated to ensure sufficient skin bridge to avoid wound healing complication [20]. Variables such as the surgical approach, age, and gender were found to influence the microcirculation of the lateral hindfoot and those need to be considered in the decision-making process as well [7].

There is a number of limitations to this study. First, we only included patients whose talar fracture underwent operative fixation. Minimally displaced talus fractures that were treated conservatively may not necessarily have the same incidence of peroneal tendon dislocation.

Secondly, CT scans may not always show peroneal dislocation; however, we reviewed the operative reports for peroneal tendon dislocation and two dislocations have been identified intra-op despite inconclusive CT scans. Although MRI evaluation of peroneal tendons might be superior to CT scans, it is not readily available for all cases and needs prior arrangement. Moreover, it leads to additional costs and delays in surgical management. Routine MRI for all talus fractures is not feasible; hence, we relied on CT scans that are usually requested for displaced talar fractures at our institute, are more cost effective, and can be done in the presence of an MRI-incompatible external fixator. Another limitation is that functional outcomes of those cases with peroneal tendon dislocation were not uniformly recorded, which precludes comparison to those with talus fractures with no concurrent peroneal tendon dislocation. However, morbidity associated with isolated peroneal tendon dislocation has been described in literature and touched upon earlier in this discussion [17–19].

Lastly, the authors acknowledge the retrospective nature of the study as well as the number of subjects included. We believe that more patients could have been included whose data were lost when migrating from paper-based to EMR a few years back, not to mention the rarity of talus fracture. However, this study has the most significant number of subjects reported in the literature so far [21–23].

## Conclusion

Peroneal tendon dislocation is associated with as high as 20% of talus fractures. The authors recommend carefully reviewing CT scans by surgeons and radiologists alike to avoid missing such injury and allow for appropriate surgical approach utilization.

The Fleck sign is a highly specific radiographic sign that has a statistically significant correlation with PT dislocation; hence, we recommend intra-operative assessment of peroneal tendons in patients with a fleck sign.
